# Arterial Hypertension and other risk factors associated with cardiovascular diseases among adults^1^

**DOI:** 10.1590/0104-1169.3345.2450

**Published:** 2014

**Authors:** Cremilde Aparecida Trindade Radovanovic, Lucimary Afonso dos Santos, Maria Dalva de Barros Carvalho, Sonia Silva Marcon

**Affiliations:** 2 PhD, Adjunct Profesor, Universidade Estadual de Maringá, Maringá, PR, Brazil; 3 PhD, Adjunct Professor, Universidade Estadual do Paraná, Paranavaí, PR, Brazil; 4 PhD, Associate Professor, Universidade Estadual de Maringá, Maringá, PR, Brazil; 5 PhD, Full Professor, Universidade Estadual de Maringá, Maringá, PR, Brazil

**Keywords:** Hypertension, Cardiovascular Diseases, Risk Factors, Adults

## Abstract

**OBJECTIVE:**

to identify the prevalence of arterial hypertension and its association with cardiovascular risk factors among adults.

**METHOD:**

cross-sectional, population-based, descriptive study conducted with 408 adult individuals. Data were collected through a questionnaire and measurements of weight, height and waist circumference. Person's Chi-square and multiple logistic regression were used in the data analysis.

**RESULTS:**

23.03% of the individuals reported hypertension with a higher prevalence among women. Odds Ratio indicated that smoking, body mass index, waist circumference, diabetes mellitus and dyslipidemia were positively associated with arterial hypertension.

**CONCLUSION:**

high self-reported hypertension and its association with other cardiovascular risk factors such as diabetes, obesity and dyslipidemia show the need for specific nursing interventions and the implementation of protocols focused on minimizing complications arising from hypertension, as well as to prevent the emergence of other cardiovascular diseases.

## Introduction

Cardiovascular diseases (CVD) are currently the leading cause of deaths worldwide. These diseases accounted for more than 17 million deaths in 2008, three million of which occurred before the age of 60. Most could have been avoided. The World Health Organization estimates that around 23.6 million people will die of cardiovascular diseases in 2030^(^ 1 ^)^.

Among the CVD, systemic arterial hypertension (SAH) is an important risk factor for cardiac and cerebrovascular complications^(^ 1 ^)^. It is considered a public health problem around the world. In 2000, the prevalence of SAH in the world population was 25% and the estimate for 2025 is 29%^(^ 2 ^)^. The prevalence of hypertension verified in studies conducted in Brazil ranged from 22.3% to 43.9%, with an average of 32.5%^(^ 3 ^-^ 4 ^)^.

In virtually all nations, the prevention and control of SAH has important implications and the use of new strategies and approaches to identify with greater accuracy the individuals at risk benefit both the individuals with hypertension and society^(^ 5 ^)^. Because SAH is a chronic disease, however, it requires monitoring and treatment for life, involving both pharmacological and non-pharmacological measures^(^ 6 ^)^.

 Considering the high levels of morbidity and mortality caused by cardiovascular diseases in Brazil and in the world, and the prevalence of arterial hypertension coupled with a scarcity of studies addressing this topic in small towns in the Southern region of Brazil, this study's aim was to identify the prevalence of arterial hypertension and its association with cardiovascular risk factors among adult individuals in Paiçandu, PS, Brazil. 

## Method

This cross-sectional, population-based, descriptive study was conducted with adult individuals living in Paiçandu, PR, Brazil. This town has a total area of 170.64 km^2^ and an estimated population of 35,941 inhabitants, 19,776 of which are adults aged between 20 and 59 years old. 

To define the sample size, a prevalence of 50% of cardiovascular risk factors was considered together with an estimated error of 5% and reliability and precision of the sample is set at 95%, adding 10% for potential losses, resulting in 415 individuals. A total of 408 individuals aged between 20 and 59 years old, both sexes, participated in this study, while only valid data were considered for some variables. 

The individuals were selected in a systematic random sample in which the streets, blocks and households were randomly drawn. Systematically and with a predefined interval, one individual living in the fourth household on the right side of the street was addressed. When no individuals within the selected age group lived in the house, we moved to the subsequent house in which only one individual was interviewed.

Data were collected from September 2010 to February 2011 through interviews and measurements of weight, height, and waist circumference (WC). The instrument used for the interviews is part of an instrument adopted in a household survey addressing behavioral risk and morbidity of non-communicable diseases^(^ 7 ^)^. This questionnaire was previously tested in a pilot-study with 20 individuals living near the campus of the State University of Maringá, Maringá, PR, Brazil. Small corrections were implemented in the instrument after the pilot-study to improve the level of understanding it provided. 

SAH was considered for self-reported cases. Socio-demographic variables were the predictor variables of sex, age, marital status, and economic status, which was used to estimate the purchasing power of individuals and families^(^ 8 ^)^, who were grouped into: A1-A2, B1-B2, C1-C2, and D-E. Variables regarding lifestyle included: smoking, diet and exercise. Smoking referred to individuals smoking regularly regardless of the number of cigarettes; individuals who did not exercise at least three times a week for at least 30 minutes per session were considered to be inactive^(^ 9 ^)^. Diet was considered inadequate when the intake of fruits and/or vegetables and/or legumes was below five times a week^(^ 7 ^)^.

Body mass (in kilograms) was assessed using a portable anthropometric digital scale (150 kilograms maximum capacity and accuracy to 0.1 kilograms). An anthropometric tape was used to measure height (in meters). The individual's weight (in kilograms) divided by the squared height (in meters) was used to compute body mass index (BMI). The values were classified into: normal weight, when BMI >18.50 to 24.99 kg/m^2^; overweight, when BMI ≥25 to 29.99 kg/m^2^; and obese when BMI ≥30 kg/m^2(9)^. Abdominal obesity (excessive fat in the abdominal region) was determined when WC was above 102 cm for men and 88 cm for women^(^ 9 ^)^, measured at the midpoint between the costal margin and the iliac crest.

The outcome variables were self-reported morbidities, such as diabetes mellitus (DM) and dyslipidemia. To verify the presence of an agglomeration of risk factors, scores ranged from none (0) to five (5) or more risk factors. Zero was scored when there was no exposure to any factors, 1 - when there was exposure to one factor, 2 - exposure to two risk factors, 3 - exposure to three factors, 4 - exposure to four factors, and 5 - exposure to five or more risk factors. Data were recorded in a database in Microsoft Office Excel 2007; double entry was used to check later for inconsistencies, and data analysis was performed using the R Environment for Statistical computing. The statistical analysis used to identify the Odds Ratio (OR) was univariate analysis considering hypertension in each of the variables (sex, age, economic status, marital status, exercise, diet, smoking, cholesterol, BMI, WC, and DM). Multiple logistic regression was performed using SAH as the outcome and all the variables were considered. Afterwards, the model was reassessed and only the variables significant at 5% were kept. Person's Chi-square test was used to verify association with the agglomeration of cardiovascular risk factors.

The study was approved by the Institutional Review Board at the State University of Maringá (COPEP-UEM), process No. 173/2010. All the participants signed two copies of free and informed consent forms.

## Results

Most of the 408 adults assessed were women (68.63%). The prevalence of SAH was 23.03%, higher among women (24.64%) than among men (19.53%), though with no significant differences (Table 1). Average age was 39.9 years old ±12 years, while 47.71% of the individuals were aged between 50 and 59 years old. The prevalent economic status was D-E, with 31.82% of the individuals being hypertensive (Table 1).

**Table 1:** Prevalence of arterial hypertension according to socio-demographic profile. Paiçandu, PR, Brazil, 2011

Variable*		Total	SAH prevalence		Raw OR (CI95%)	P-value
			n	%		
Sex (n=408)						0.311
	Female	280	69	24.64	1	
	Male	128	25	19.53	0.77 (0.45-1.27)	
Age groups – years (n=408)						<0.001
	20 to 29	100	09	9.00	1	
	30 to 39	94	14	14.89	1.71 (0.71-4.29)	
	40 to 49	105	19	18.10	2.21 (0.97-5.37)	
	50 to 59	109	52	47.71	8.76 (4.19-20.23)	
Economic status (n=399)						0.014
	A1-A2; B1-B2	129	20	15.75	1	
	C1-C2	248	67	27.02	1.99 (1.16-3.53)	
	D-E	22	07	31.82	2.26 (0.79-6.01)	
Marital status (n=407)						0.961
	No partner	132	30	22.73	1	
	Partner	275	63	22.91	0.99 (0.61-1.63)	

* Only valid data were considered

Table 2 shows that the factors most frequently found among hypertensive individuals were DM, obesity and dyslipidemia.

**Table 2:** Prevalence of arterial hypertension according to cardiovascular risk factors and self-reported morbidities. Paiçandu, PR, Brazil, 2011

Variables*		Total	Prevalence of SAH		Raw Odds Ratio (CI 95%)	P-value
			n	%		
Exercise (n=407)						0.542
	Yes	90	23	25.56	1	
	No	317	71	22.40	1.18 (0.68-2.01)	
Diet (n=403)						0.648
	Appropriate	315	74	23.49	1	
	Inappropriate	88	19	21.59	0.88 (0.49-1.52)	
Smoking (n=407)						
	Non-smoker	272	48	17.65	1	<0.001
	Smoker	66	18	27.27	1.73 (0.91-3.18)	
	Former smoker	69	28	40.58	3.27 (1.84-5.80)	
BMI (n=405)						<0.001
	Normal	172	27	15.70	1	
	Overweight	144	34	23.61	1.71 (0.98-3.02)	
	Obese	89	33	37.08	3.13 (1.74-5.69)	
Waist circumference (n=396)						0.008
	Normal	116	18	15.52	1	
	Greater than normal parameters	280	75	26.79	2.12 (1.24-3.76)	
Diabetes Mellitus (n=408)						<0.001
	No	376	78	20.74	1	
	Yes	32	16	50.00	3.91 (1.86-8.22)	
Dyslipidemia (n=408)						<0.001
	No	330	64	19.39	1	
	Yes	78	30	38.46	2.6 (1.52-4.41)	

* Only valid data were considered

Significant association was found between SAH and smoking (p<0.001), obesity (p<0.001), WC (p=0.022), DM (p<0.001), and dyslipidemia (p<0.001). The variables exercise and diet presented no significant differences (p=0.542, p=0.648, respectively).

After adjusting the variables, the presence of SAH was statistically significant for age, smoking, BMI, and DM, as presented in Table 3.

**Table 3:** Factors associated with arterial hypertension according to multiple regression analysis. Paiçandu, PR, Brazil, 2011

Variable		Odds Ratio	CI (95%)	P-value
Age group – years				
	20 to 29	1	-	-
	30 to 39	1.23	0.49-3.22	0.655
	40 to 49	1.58	0.66-3.98	0.306
	50 to 59	5.35	2.41-12.91	<0.001
Smoking				
	Non-smoker	1	-	-
	Smoker	2.36	1.13-4.83	0.019
	Former smoker	2.21	1.16-4.18	0.014
BMI				
	Normal	1	-	-
	Overweight	1.27	0.67-2.43	0.454
	Obese	2.35	1.19-4.64	0.013
Diabetes Mellitus				
	No	1	-	-
	Yes	2.89	1.27-6.60	0.010

Individuals aged between 50 and 59 years old were 5.35 times more likely to present hypertension compared to those aged from 20 to 29 years old. Smokers are 2.36 times more likely than non-smokers; obese individuals were 2.35 times more likely than those within normal parameters of weight, while individuals with DM are 2.9 times more likely to be hypertensive than individuals without DM.

Table 4 shows that 40.38% of the hypertensive individuals aged from 50 to 59 years old have five or more cardiovascular risk factors, while 25% of the hypertensive individuals aged between 20 to 29 years old do not present any cardiovascular risk factors. A total of 33.33% of the hypertensive individuals aged between 30 and 39 years old presented two risk factors. 

**Table 4:** Distribution of hypertensive individuals according to the number of cardiovascular risk factors. Paiçandu, PR, Brazil, 2011

Risk factors	Age groups (years)										
	20 to 29			30 to 39			40 to 49			50 to 59	
	%	CI 95%		%	CI 95%		%	CI 95%		%	CI 95%
0	25.00	(4.45-64.42)		8.33	(0.44-40.25)		10.53	(1.84-34.54)		0.00	(0.00-8.57)
1	25.00	(4.45-64.42)		16.67	(2.94-49.12)		21.05	(6.97-46.10)		3.85	(0.67-14.33)
2	12.50	(0.66-53.32)		33.33	(11.27-64.56)		21.05	(6.97-46.10)		17.31	(8.69-30.82)
3	12.50	(0.66-53.32)		25.00	(6.69-57.16)		10.53	(1.84-34.54)		17.31	(8.69-0.82)
4	25.00	(4.45-64.42)		8.33	(0.44-40.25)		10.53	(1.84-34.54)		21.15	(11.52-35.09)
≥5	0.00	(0.00-40.23)		8.33	(0.44-40.25)		26.32	(10.12-51.42)		40.38	(27.31-54.87)

P-value=0.0385

## Discussion

The prevalence of SAH identified in this study was similar to that found in a study conducted in Pelotas, in the Southern region of Brazil^(^ 10 ^)^, and less than that observed in other Brazilian studies^(^ 4 ^,^ 11 ^)^ and in studies conducted in the United States^(^ 12 ^)^. It is, however, greater than the prevalence identified in a more comprehensive study conducted in all the Brazilian capitals using self-reported information, which ranged from 14.7% (in Palmas) to 29.5% (Rio de Janeiro), with an average of 21.6%^(^ 13 ^)^. Hypertension was more frequently found among women, which corroborates the findings of other studies conducted with adult^(^ 4 ^,^ 13 ^-^ 14 ^)^ and elderly individuals^(^ 4 ^,^ 13 ^-^ 15 ^)^, though no statistically significant association was observed between sexes, confirming the results reported for Nobres, MT^(^ 4 ^)^ and Firminópolis, GO, Brazil^(^ 11 ^)^.

This study also shows that the prevalence of hypertension increased with age, as reported by another study^(^ 16 ^)^, while individuals aged between 50 and 59 years old were 5.35 times more likely to present hypertension that individuals aged from 20 to 29 years old.

The variable exercise was not significantly associated with SAH, similar to the results found in one study conducted in Sinop, MT, Brazil, though the authors report that longitudinal studies are more appropriate to assess the effects of exercise on blood pressure^(^ 17 ^)^. Regular exercise is a non-pharmacological measure recommended for the treatment of SAH, not only for its beneficial effect on blood pressure, but also to reduce other cardiovascular risk factors^(^ 18 ^)^.

Likewise, diet was not significantly associated with SAH. Such a result may be associated with the type of methodology employed to assess diet. Only the intake of vegetables, greens and fruits was considered, though the intake of salt and fat is also an important indicator of SAH. Appropriate food intake and the importance of a balanced diet should be stressed and recommended by health professionals with hypertensive patients^(^ 14 ^)^ for its benefit of reducing cardiovascular risks. 

In regard to smoking, former smokers presented the highest prevalence of hypertension, which corroborates the findings of other studies^(^ 10 ^-^ 11 ^)^. This study also shows that former smokers and smokers presented significant association with SAH. One study conducted in Japan reports that smoking and SAH are the two main factors of mortality due to non-communicable diseases among adults^(^ 19 ^)^.

This study found significant association in regard to BMI, similar to the findings of other studies that also show a significant association with SAH^(^ 20 ^-^ 21 ^)^. The obese individuals in this study were 2.35 times more likely to present hypertension compared to those within a weight range considered to be normal. WC also was an important anthropometric indicator, showing significant association with SAH, corroborating the results reported by a study addressing Chinese individuals^(^ 22 ^)^.

The prevalence of obesity has grown around the world and is considered an important risk factor for SAH. One study conducted in the northern part of China reports that increased BMI is strongly linked to hypertension; however, it only represents the total body weight and is unable to distinguish between excess fat tissue and a large amount of muscle mass^(^ 22 ^)^.

This study shows that individuals with DM are almost three times more likely to develop SAH than individuals without DM. One epidemiological study indicates that DM and hypertension are usually associated conditions^(^ 16 ^)^, confirming data found in this study, that is, that 50% of the individuals with DM also have hypertension. Self-reported dyslipidemia also was significantly associated with SAH, though this association did not remain after the variable was adjusted. High levels of cholesterol associated with hypertension account for more than 50% of the risk attributed to coronary disease, so that therapeutic measures can reduce morbidity and mortality in diverse conditions of risk^(^ 23 ^)^. We also observed that most individuals with SAH presented more than three cardiovascular risk factors. Additionally, an increase in the number of associated factors was maintained with aging.

This study's limitations are related to three aspects: a) the fact that the participants were selected according to which individuals were present at home at the time of the visit, which resulted in a sample predominantly composed of women; b) SAH was self-reported; and c) the fact that leisure activities were not considered exercise, which led to a high prevalence of sedentariness. In regard to the last two questions, however, it is important to note that self-reported hypertension is considered appropriate to estimating the prevalence of various health conditions in populations and has also been used by the Ministry of Health in the VIGITEL^(^ 13 ^)^ survey. Also, in regard to exercise, we note that an evaluation of a population as being sedentary should generally be viewed with caution, especially when making journeys, domestic and work activities are not taken into account^(^ 24 ^)^. 

## Conclusion

The results show that the prevalence of self-reported SAH was higher among women aged between 50 and 59 years old. In regard to risk factors, the most prevalent were DM, obesity, and dyslipidemias, while these are considered important risk factors for arterial hypertension. 

The identification of SAH and its association with other cardiovascular risk factors enabled understanding the health profile of the population under study and aided in the identification of the urgent need for specific nursing interventions, the implementation of protocols focused on minimizing complications arising from arterial hypertension, as well as efforts to prevent the emergence of other cardiovascular diseases. These interventions should be implemented in such a way they enables individuals to discuss issues concerning their chronic conditions and associated risk factors, while at the same time, providing encouragement and support in the promotion of a healthier lifestyle. In this sense, it is highly recommended that nursing professionals and other health professionals, involved with interventions, appreciate the individuals' experiences, knowledge and limitations, and implement individualized actions to provide conditions for effective behavioral changes to occur. 

Finally, even though the results obtained here involve only a small town, they show that the number of adult individuals self-reporting hypertension was high, which does not differ from what has been observed in other Brazilian cities of different population sizes, as previously noted. Additionally, the risk factors associated with hypertension identified in this town show that these issues represent a challenge for the health sector in small towns, as well, because these reflect modern lifestyles, such as smoking, inappropriate diet, and sedentariness. These habits result in health problems such as DM, obesity, and dyslipidemia, which, in this study presented significant associations with SAH, an important cause of morbidity and mortality in adults in Brazil and in the world. These findings reinforce the need for further studies comparing lifestyles and behaviors in health with the presence of cardiovascular risk factors in populations living in small and large cities.
